# Effects of *n*-3 very-long-chain PUFA on bone mineralisation

**DOI:** 10.1017/S0007114524003052

**Published:** 2025-01-28

**Authors:** Martina Torrissen, Elisabeth Ytteborg, Harald Svensen, Tone-Kari Østbye, Gerd Berge, Sjur Reppe, Aleksei Krasnov, Bente Ruyter

**Affiliations:** 1 Nofima (Norwegian Institute of Food, Fisheries and Aquaculture Research), Ås 1432, Norway; 2 Epax Norway, Ålesund 6006, Norway; 3 NMBU (Norwegian University of Life Sciences), Ås 1433, Norway; 4 Department of Medical Biochemistry, Oslo University Hospital, Oslo 9424, Norway; 5 Department of Plastic and Reconstructive Surgery, Oslo University Hospital, Oslo, Norway; 6 Unger-Vetlesen Institute, Lovisenberg Diaconal Hospital, Oslo, Norway

**Keywords:** Very-long-chain PUFA, *n*-3 fatty acids, Bone, Mineralisation

## Abstract

The present study investigated whether dietary *n*-3 very-long-chain PUFA (*n*-3 VLC-PUFA) could increase skin and bone mineralisation in Atlantic salmon (*Salmo salar*) *in vivo* and examined their potential effects on human osteoblast proliferation and differentiation *in vitro*. Atlantic salmon were fed different dietary levels of *n*-3 VLC-PUFA, and changes in tissue *n*-3 VLC-PUFA composition, skeletal morphology, skin and bone mineral content, bone mineral density (BMD) and gene expression patterns were examined. Additionally, *in vitro* experiments using human foetal osteoblast cells were conducted to investigate the potential influence of *n*-3 VLC-PUFA supplementation on cell proliferation, osteogenic differentiation and cytokine expression. The results demonstrated that increasing the dietary levels of *n*-3 VLC-PUFA increased the mineral content of vertebrae and BMD in salmon, with subtle yet significant impacts on the expression of genes involved in bone-related processes. Furthermore, *in vitro* experiments showed a potential contextual influence of *n*-3 VLC-PUFA supplementation on gene expression of osteogenic markers and cytokine expression. Our findings indicate for the first time that *n*-3 VLC-PUFA may influence processes related to bone mineralisation.

Osteoporosis is a major health problem in post-menopausal women and in the elderly population^(1)^. It may cause morbidity and mortality, and an economic burden on society^(1)^. While there are therapies to treat osteoporosis, of which bisphosphonates, recombinant human parathyroid hormone and humanised antibodies are the most effective^(2)^, they may have adverse side effects^(3,4)^. Diets and lifestyles that minimise bone loss are therefore preferred to reduce and prevent osteoporosis, as well as the side effects caused by these therapies.

Increased consumption of dietary *n*-3 PUFA has been shown to increase bone strength via different mechanisms in different species. In post-menopausal mice, *n*-3 fatty acids have been shown to protect against bone loss^(5–7)^. Furthermore, *n*-3 fatty acids from oily fish have been shown to decrease levels of cytokines, such as IL-1*β*, IL-5 and TNF-*α*, and reduce bone resorption^(8)^. Consumption of *n*-3 fatty acids has further been shown to protect against age-related bone loss, and high intake of oily fish, rich in *n*-3 fatty acids, has been associated with greater bone mass and lower risk of osteoporosis in post-menopausal women^(9)^. In a study conducted by Lage *et al.* ^(10)^, a lack of essential *n*-3 fatty acids demonstrated potential effects on bone mineralisation, and a comprehensive review by Pino and Rodríguez^(11)^ highlighted the relevance of fatty acid composition in bone health. Additionally, several experimental studies have revealed that *n*-3 fatty acids promote mineral deposition in bone and provide strength to the bone by inhibiting bone resorption with a potential reversal of bone loss^(12)^. Furthermore, in Atlantic salmon (*Salmo salar*), unspecialised precursor cells showed improved differentiation *in vitro* when supplemented with *n*-3 PUFA^(13)^. On the other hand, excess consumption of *n*-6 fatty acids may have a negative effect on bone formation by promoting the differentiation of mesenchymal stem cells to adipocytes instead of osteoblasts, which is a main cause of age-related bone loss^(14)^.

While *n*-3 PUFA have been extensively studied for their various roles in physiological processes, *n*-3 very-long chain PUFA (VLC-PUFA), defined as fatty acids with a carbon chain length of ≥ 24, are less well understood. These fatty acids are characterised by their longer carbon chains, but are distinct from the well-studied *n*-3 PUFA eicosapentaenoic acid (EPA) and DHA^(15)^. Several studies have suggested that VLC-PUFA may have specific functions in various tissues, as evidenced by different pathologies when they are absent^(16)^. However, the exact nature of these functions remains unclear^(15–19)^.

We previously reported that dietary supplementation with an *n*-3 VLC-PUFA concentrate in the early life stages of Atlantic salmon increased mineralisation and maturation of the scales of the skin. The scale development bears many similarities to the development of bone tissue in vertebrates, including mammals^(20)^. In fact, there are several physiological and morphological features shared between teleost scales and mammalian and fish bone^(21)^, sharing several matrix proteins such as type I collagen, bone *γ*-carboxyglutamic acid protein and osteonectin^(21–23)^. The cells osteoblasts and osteoclasts, responsible for building the bone matrix in both mammals and fish, are also shown to have similar functions in both species. Studies of processes related to bone development in Atlantic salmon can thereby provide important knowledge of relevance for human research on bone development^(21,24)^. Furthermore, Elovl4-like elongase in Atlantic salmon has been identified as a critical enzyme in the biosynthesis of very-long-chain fatty acids (VLC-FAs), with mRNA transcripts abundant in the eye, brain and testes – similar to what is observed in mammals^(25)^. This further underscores the relevance of Atlantic salmon as a model for studying VLC-PUFA and their potential roles in bone development, with implications for understanding similar processes in humans.

The present study investigated the potential of *n*-3 VLC-PUFA to influence bone mineralisation following a 5-week dose-response feeding trial in Atlantic salmon and the effects of *n*-3 VLC-PUFA on the proliferation and differentiation of the human foetal osteoblast cell line, hFOB 1·19. Both the fish diets and growth media were designed to include increasing levels of VLC-PUFA to capture changes related to bone development, such as stimulation of cell proliferation, osteogenic differentiation, and mineralisation. Several studies have shown associations between the consumption of LC-*n*-3 fatty acids and beneficial effects on bone health; however, to our knowledge, no studies have investigated the effects of the novel *n*-3 VLC-PUFA on bone health.

## Materials and methods

### Production of *n*-3 VLC-PUFA concentrates

Two different *n*-3 VLC-PUFA concentrates were made from an anchovy fish oil distillation fraction by hydrolysis, precipitation with LiOH and distillation^(26)^ as previously described^(27)^. Both concentrates were produced by Epax Norway AS (Aalesund, Norway). The fatty acid composition of the concentrates used in the salmon feeding trial and cell culture experiments are presented in Table 1. In the feeding trial and cell culture experiments, different concentrates were used to isolate the effects of *n*-3 VLC-PUFA from those of other fatty acids and to increase the concentration of *n*-3 VLC-PUFA, allowing for the addition of smaller volumes in the *in vitro* studies. In the feeding trial, the salmon diet already contained a variety of other fatty acids, which meant there was no need for a concentrate devoid of SFA, ≤ 22-carbon MUFA, and PUFA. Consequently, the composition of the concentrate used in the feeding trial differed from that used in the cell culture experiments, which required a more specific formulation to accurately assess the effects of *n*-3 VLC-PUFA in a controlled environment.

**Table 1.** Fatty acid composition of VLC-PUFA concentrates^*^

	VLC-PUFA concentrate in salmon feed (%)	VLC-PUFA concentrate in cell culture trials (%)
26:0	0·19	nd
Sum of SFAs	0·19	nd
22:1 *n*-9	0·33	nd
22:1 *n*-11	3·37	nd
24:1 *n*-9	2·69	1·29
26:1 *n*-9	0·20	2·97
Sum of MUFAs	6·59	4·26
22:5 *n*-6	0·37	nd
Sum of *n*-6 PUFA	0·37	nd
18:4 *n*-3	0·20	nd
20:5 *n*-3	0·34	nd
22:5 *n*-3	4·30	0·20
22:6 *n*-3	13·29	0·68
Sum of *n*-3 LC-PUFA	18·13	0·88
24:4 *n*-3	1·25	0·33
24:5 *n*-3	6·57	1·47
24:6 *n*-3	3·02	0·47
26:3 *n*-3	0·20	nd
26:4 *n*-3	1·97	nd
26:5 *n*-3	3·05	4·34
26:6 *n*-3	7·24	9·56
26:7 *n*-3	1·07	0·95
28:5 *n*-3	0·71	2·04
28:6 *n*-3	2·59	5·31
28:7 *n*-3	0·93	1·42
28:8 *n*-3	31·31	62·25
32:8 *n*-3	0·28	nd
Sum of 30:5, 30:6, 30:8 fatty acids	2·53	5·03
Sum of *n*-3 VLC-PUFA	62·72	93·17
Sum of VLC-FAs	65·61	97·43
Total	88·27	98·31

ND, not detected; SFAs, saturated fatty acids; PUFA, polyunsaturated fatty acids; VLC-PUFA, very-long-chain PUFA.

Data are presented as percentage of total fatty acids.

* The analytical methods used detect fatty acids ≤ C30.

### Atlantic salmon feeding trial

Atlantic salmon ((SAL-1–22), Salmo Breed) were housed in freshwater tanks (100-L, *n* 100 fish per tank) at Nofima’s Aquaculture research station for sustainable aquaculture at Sunndalsøra, Norway. The fish were maintained in conditions of 24 h light and temperature controlled to 12·5°C (±0·5°C). The water flow was set to 5 l/min, and oxygen was adjusted to 85 %–100 % by adding oxygen to the water holding tank when needed. Mortality was recorded daily and the trial lasted 5 weeks.

One experimental extruded basal diet without lipid addition was produced at the Nofima’s aquafeed technology centre (Bergen, Norway), with a pellet size of 1·5 mm (similar to a method previously described by Bogevik *et al.*, 2021^(28)^). The basal diet added the inert mineral marker yttrium which is not digested. This marker enables analyses of mineral digestibility/uptake in intestine according to the method described by Hatlen *et al.*, 2015^(29)^. The basal diet was then divided into five equal batches. The five diets were made by coating each extruded basal feed batch with five different oil mixes (vacuum coater, Dinnisen BV, Sevenum, the Netherlands) to give the same total fat content of all diet groups (Table 2).

**Table 2.** Ingredient composition and PUFA content of the basal diet

	Diet groups				
	Ctr	L-VLC	I-VLC	MH-VLC	H-VLC
Composition of basal mix (%)					
Fish meal^*^	50·00	50·00	50·00	50·00	50·00
Soy protein concentrate^†^	10·00	10·00	10·00	10·00	10·00
Wheat gluten^‡^	9·00	9·00	9·00	9·00	9·00
Wheat^§^	13·11	13·11	13·11	13·11	13·11
Choline chloride^║^	0·50	0·50	0·50	0·50	0·50
Canola lecithin^¶^	0·50	0·50	0·50	0·50	0·50
Microingredients^**^	3·01	3·01	3·01	3·01	3·01
Water adjustment	–0·32	–0·32	–0·32	–0·32	–0·32
Oils coated onto the basal diet mix					
Fish oil (Herring oil, Nofima)^††^	13·83	13·04	12·25	11·45	10·66
VLC-PUFA^‡‡^		0·80	1·59	2·39	3·18
EPA/DHA (EPAX 6015 TGN)^‡‡^		0·09	0·18	0·27	0·36
EPA/DHA (EPAX 0460 TGN)^‡‡^	0·37	0·28	0·19	0·09	
SUM	100·00	100·00	100·00	100·00	100·00
LC-PUFA and VLC-PUFA content					
EPA, % of diet	1·17	1·17	1·17	1·17	1·17
DHA, % of diet	1·65	1·65	1·65	1·65	1·65
EPA + DHA, % of diet	2·82	2·82	2·82	2·82	2·82
EPA + DHA, % of fatty acids	14·85	14·85	14·85	14·85	14·85
VLC-PUFA, % of diet	0·00	0·47	0·95	1·42	1·90
VLC-PUFA, % of fatty acids	0·00	2·50	5·00	7·50	10·00

Ctr, Control; L-VLC, Low-VLC; I-VLC, Intermediate-VLC; MH-VLC, Medium-High-VLC; H-VLC, High-VLC.

* Vedde AS (Norway).

† Agrokorn (Germany).

‡ Tereos Syrdal (France).

§ Norgesmøllene AS (Norway).

║ Vilomix (Norway).

¶ Marvesa Oil and Fats (Netherland).

** Normin (Norway), Provided per 100 g of feed: K, 80 mg; Mg, 75 mg; Zn, 12 mg; Fe, 6 mg; Mn, 3 mg; Cu, 0·6 mg; Se, 0·03 mg; vitamin D, 300 mg; vitamin E, 16 mg; thiamine, 2 mg; riboflavin, 3 mg; pyridoxine-HCl, 3 mg; vitamin C, 20 mg; calcium d-pantothenate, 6 mg; biotin, 0·11 mg; folic acid, 1 mg; niacin, 20·1 mg; cobalamin, 0·005 mg; vitamin K3, 2 mg.

†† Pelagia (Norway).

‡‡ Epax Norway AS (Norway).

The oil mix of the different diets contained different levels of VLC-PUFA (0 %, 2·5 %, 5 %, 7·5 % and 10 % of total fatty acids, named Control, Low-VLC (L-VLC), Intermediate-VLC (I-VLC), Medium-High-VLC (MH-VLC) and High-VLC (H-VLC), respectively), and levels of EPA and DHA were kept constant at 14·9 % of the total fatty acids between all the groups. The amount of fish oil was reduced from 18·83 % to 10·66 % as the amount of VLC-PUFA oil included in the diets increased. Each diet was fed to triplicate tanks. The total ingredient composition and PUFA content of the diets are presented in Table 2.

The weights of all the experimental fish in each tank were recorded in bulk at the start of the experiment and after 5 weeks, and growth rates were calculated based on the average weight of the fish in each tank, following the formula described in^(27)^.

#### Fatty acid composition of Atlantic salmon feed

The fatty acid composition of the experimental salmon diets is presented in Table 3. There was a linear increase in the sum of VLC-PUFA in the feed, in line with the intended fatty acid composition of the diets. Furthermore, the sum of EPA (20:5 *n*-3) and DHA (22:6 *n*-3) content was approximately the same in all the diet groups. The fatty acid composition of the salmon feed, presented as percentage of total fatty acids, is presented in online Supplementary Table S1.

**Table 3.** Fatty acid composition of the experimental salmon diets

	Diet groups				
	Ctr	L-VLC	I-VLC	MH-VLC	H-VLC
14:0	10·74	9·71	10·54	9·00	8·84
15:0	0·20	0·19	nd	0·63	0·18
16:0	22·46	20·69	22·83	19·97	19·60
17:0	0·33	0·29	1·33	0·29	0·25
18:0	2·57	2·40	2·42	2·25	2·22
20:0	0·29	nd	0·26	0·26	0·25
24:0	0·31	0·31	0·30	0·34	0·32
Sum of SFAs	36·90	33·59	37·69	32·73	31·66
14:1 *n*-5	0·57	0·58	1·35	0·56	0·45
15:1	0·35	0·31	0·36	0·34	0·27
16:1 *n*-7	8·28	6·90	7·48	6·61	7·00
16:1 *n*-5	0·75	0·68	0·85	0·68	0·20
17:1 *n*-7	0·60	0·55	0·79	0·55	0·47
18:1 *n*-7	3·12	2·97	3·10	2·86	2·82
18:1 *n*-9	21·27	19·34	20·12	18·65	18·36
18:1 *n*-11	0·81	0·14	0·75	0·68	0·35
19:1	0·26	0·20	0·24	0·24	0·23
20:1 *n*-7	19·09	17·66	16·95	16·00	17·21
20:1 *n*-11	4·14	3·82	4·06	3·71	3·65
22:1 *n*-7	0·88	0·82	0·85	0·80	0·78
22:1 *n*-9	3·19	3·10	1·54	2·50	2·54
22:1 *n*-11	28·58	26·33	26·32	24·58	24·22
24:1 *n*-9	1·74	1·74	1·99	1·99	1·98
Sum of MUFAs	93·63	85·13	86·75	80·77	80·54
16:2 *n*-6	0·62	0·55	0·54	0·55	0·52
18:2 *n*-6	7·18	6·77	7·82	7·43	7·32
18:3 *n*-6	0·24	0·22	0·24	0·20	0·20
20:2 *n*-6	0·38	0·34	0·36	0·32	0·32
20:4 *n*-6	0·55	0·55	0·62	0·60	0·60
22:4 *n*-6	0·57	0·53	0·54	0·48	0·48
Sum of *n*-6 PUFA	9·55	8·95	10·13	9·57	9·44
16:2 *n*-3	0·57	0·51	0·53	0·48	0·40
20:4 *n*-3	1·14	0·78	2·22	1·43	nd
20:5 *n*-3	9·84	9·31	12·44	10·78	10·51
22:5 *n*-3	1·70	1·79	2·07	2·18	2·15
22:6 *n*-3	16·45	15·66	16·16	15·66	15·41
Sum of *n*-3 PUFA	31·85	30·01	35·55	32·49	30·40
Sum EPA/DHA	26·29	24·97	28·60	26·44	25·92
24:4 *n*-3	0·09	0·25	0·23	0·28	0·33
24:5 *n*-3	0·57	0·87	1·30	1·71	2·05
24:6 *n*-3	0·17	0·33	0·52	0·73	0·91
26:4 *n*-3	0·06	0·15	0·30	0·43	0·52
26:5 *n*-3	0·04	0·20	0·40	0·62	0·76
26:6 *n*-3	0·05	0·43	0·88	1·37	1·75
28:8 *n*-3	0·17	1·70	3·44	5·56	6·88
Sum of *n*-3 VLC-PUFA	1·18	3·99	7·22	10·92	13·48
Fat %	21·99	22·46	22·54	22·50	22·29

Ctr, Control; L-VLC, Low-VLC; I-VLC, Intermediate-VLC; MH-VLC, Medium-High-VLC; H-VLC, High-VLC; ND, not detected.

Data are presented as mg/g feed.

#### Fish sampling and sample preparation

Whole-body samples of 20 fish from each tank were frozen at the start and end of the experiment, including 10 for whole-body lipid composition analyses and 10 for X-ray analyses. At the end of the experiment, 10 fish from each tank were randomly sampled and euthanised by an overdose of the anaesthetic metacaine (MS-222; 0·05–0·08 g/l). Samples of vertebrae and skin were frozen in liquid nitrogen (Nippon gases, Norway) and then stored at –80°C for later analysis. Samples of vertebrae were stored in RNAlater (Thermo Fisher Scientific, Waltham, Massachusetts, USA). The remaining fish in each tank were stripped for faeces, which were frozen at –20°C for later analyses of mineral digestibility.

#### Mineral analysis

For mineral content analysis, sample preparation followed the method described in^(30)^. Mineral content analysis was performed on vertebrae and skin. Pooled feed and faeces samples (*n* 10) from each tank were analysed for minerals together with the inert marker yttrium in order to measure intestinal mineral uptake, and bioavailability of minerals was determined according to Austreng^(31)^.

#### Whole-Body x-ray and computed tomography (CT) analyses of vertebrae

##### Skeletal morphology

The morphology of the vertebral column and skull were examined by means of radiography at the beginning and end (*n* 5 per tank) of the experiment. Radiography was performed in a semi-digital system using a mammography X-ray source (IMS Giotto, Pontecchio Marconi, BO, Italy). X-ray images were recorded on coated photoreactive phosphorous (FCR Imaging Plates, Fujifilm, Tokyo, Japan). The image resolution was 20 pixels per mm^2^, with exposure at 22 kV and 100 mA. Plates were read using an FCR Profect Reader (Fujifilm, Tokyo, Japan). The images were evaluated with respect to any deviation of normal morphology of skeletal structures.

##### CT

The bone mineral density (BMD) measurements were done with a Skyscan 1275 micro-CT scanner (CS). The samples and phantoms were scanned with an aluminium filter and a source voltage and current at 50 kV and 200 uA, and a pixel size of 10 um. The scans were reconstructed with nrecon-reconstruction-software (nrecon, version 1.7.3), with a beam hardening correction of 55 and the min value of the CS to image conversion 0 0000–0 08000. The BMD was found with CT-analyser (CTan, v 1·20·3·0). The scans of the phantoms (0·25 g/cm^3^ and 0·75 g/cm^3^) were used for calibration. The BMD in the samples were found by selecting which part of the scans are bones and finding the average BMD in CTan.

### *In vitro* culture of human foetal osteoblast cells

An *in vitro* cell culture study using a human foetal osteoblast cell line (hFOB 1·19, ATCC, CRL-11372™)^(32)^ was used to examine the effect of different VLC-PUFA concentrations on the expression of proliferation and differentiation-related markers.

Cells were cultured according to the manufacturer’s instructions. Basal medium comprised a 1:1 mixture of Ham’s F12 Medium Dulbecco’s Modified Eagle’s Medium with 2·5 mM L-glutamine (without phenol red) (Sigma-Aldrich, Darmstadt, Germany). Complete growth medium was prepared using 0·3 mg/ml G418 (Sigma-Aldrich, Darmstadt, Germany) supplemented with 10 % foetal bovine serum (T7524, Sigma-Aldrich, Darmstadt, Germany). Cells were incubated at 34°C, with 5 % CO_2_ (In-Vitro Cell NU-8600E, NUAIRE, Minnesota, USA) and subcultured at approximately 80 % confluency at a ratio of 1:4. Growth medium was renewed every 2–3 d. Cells cultured for differentiation were supplemented with osteogenic factors, ascorbic acid (100 µg/ml; A8960, Sigma-Aldrich, Darmstadt, Germany), and *β*-glycerophosphate (10 mM; G9422, Sigma-Aldrich, Darmstadt, Germany). Once the cells reached confluency, the temperature was increased to 37°C to stop proliferation and induce differentiation. The osteogenic media was renewed every 2–3 d.

Fatty acids were added to the growth media in the form of their sodium salts bound to bovine serum albumin (Sigma-Aldrich, Darmstadt, Germany; 2·7:1 molar ratio) as previously described by Bou *et al.* ^(33)^. The pH was adjusted to 7 using NH_4_OH. *n*-3 VLC-PUFA and DHA (Sigma-Aldrich, Darmstadt, Germany) concentrates were prepared as 1 and 8-mM stocks, respectively, and were added to the media at concentrations of 1 and 2 µM. All fatty acid solutions were stored at –80°C.

For gene expression analysis of cell proliferation markers, cells were plated in 6-well plates (Thermo Fisher Scientific, Waltham, Massachusetts, USA) at passage 4–5 at a ratio of 1:3. Growth media was renewed and test substrates were added after an attachment period of 24 h. On days 2 and 3 after plating, cells were harvested for gene expression analysis. The experiment was repeated three times, with three replicates per test dose in all batches except one in which the control group had six replicates. For gene expression analysis of markers related to osteogenic differentiation, the same plating and ratio used for proliferation was used. At day 10 after inducing differentiation, the cells were supplemented with test substrates. Osteogenic growth media with and without the respective test substrates were renewed every 2–3 d. At day 7 after adding test substrates, cells were harvested for gene expression analysis, and growth media was frozen and stored at –80°C for later analysis. The experiment was repeated using three separately thawed cell batches, with six replicates per test dose in one of the experiments, five replicates in the control group, four in the 1 µM and 2 µM VLC-PUFA groups, three in the 1 µM DHA group, and two in the 2 µM DHA group in another experiment, and 11 replicates in the control group and four in each of the remaining groups in the last experiment. The separate experiments were used as an experimental unit (*n* 3), and as there were no significant differences between the two doses within the VLC-PUFA and DHA groups, the results from the 1 and 2 µM doses were combined.

For fatty acid composition analysis, cells were plated at a ratio of 1:3 from the previous passage (passage 5) in T25 tissue culture flasks (Thermo Fisher Scientific, Waltham, Massachusetts, USA). Osteogenic differentiation was induced, substrates were added as previously described, and cells were harvested for fatty acid composition on day 7 after adding the test substrates. Cells were washed twice in phosphate-buffered saline (PBS) with 1 % bovine serum albumin, then twice in PBS, and loosened in 0·5 ml of PBS by using a rubber scraper then transferred into an Eppendorf tube and centrifuged for 550 × g for 5 min. The PBS was then removed, leaving only the cell pellet in the tube, which was then frozen and stored at –80°C until further analysis.

### RNA purification and gene expression analysis

#### RNA isolation and cDNA synthesis

Cells were harvested for RNA extraction by washing twice in PBS, and then, 1 ml of QIAzol™ Lysis Reagent (Qiagen, Valencia, CA, USA) was added to the wells. Cells were then loosened using a rubber scraper and then transferred to a spin column and frozen at –80°C. Total RNA was isolated by adding 0·2 ml of chloroform (Merck, Germany) to the samples, vortexing for 15 s, and incubating for 2–3 min. The samples were then centrifuged at 12 000 × g at 4°C for 15 min. The aqueous phase was transferred to a new tube, and one volume of 95–100 % ethanol was added. RNA was then cleaned using an RNA Clean & Concentrator™-5 Kit (Zymo Research, Irvine, CA, USA) according to the manufacturer’s protocol. All samples were treated with DNase I (Invitrogen, Carlsbad, USA). The concentration and purity of RNA were evaluated using a NanoDrop 1000 Spectrophotometer (NanoDrop Technologies, USA). cDNA was synthesised using TaqMan reverse transcriptase reagents (Applied Biosystems, Foster, City, CA, USA) according to the manufacturer’s protocol.

#### qPCR

The expression of target genes was analysed using a QuantStudio Real-Time PCR analyser (Thermo Fisher Scientific, MA, USA) using two different assays. The proliferation genes *MKI67*, *MYBL2*, *CCNB1* and proliferating nuclear antigen were analysed using PowerUp SYBR Green Master Mix (Applied Biosystems, Foster City, California, USA) (Table 4), whereas runt-related transcription factor 2 (*RUNX2*), alkaline phosphatase (*ALP)*, bone gamma-carboxyglutamate protein (*BGLAP)* and collagen type I*α*1 chain (*COL1A1)* were analysed using commercially available TaqMan probes (Table 5). The specificity of the primers for the proliferation genes was confirmed by Sanger sequencing (Eurofins Genomics). The qPCR reaction mixture for the TaqMan probe assays consisted of 4 µl of a 1:10 dilution of cDNA, 0·5 µl of primer (Table 5), 0·5 µl of distilled H2O and 5 µl of TaqMan Master Mix (TaqMan™ Gene expression Master Mix, Applied Biosystems™ 4 369 514), while the SYBR Green mix contained 4 µl of a 1:10 dilution of cDNA, 1 µl of forward and reverse primers (final concentration of 0·5 µM; Table 4) and 5 µl of PowerUp SYBR Green Master Mix (Applied Biosystems, Foster City, California, USA). All samples were analysed in parallel and non-template and non-enzyme controls were included. The TaqMan-based assays were performed under the following conditions: one cycle at 50°C for 2 min; amplification for 40 cycles at 95°C for 15 s; and 60°C for 1 min. The other assays were performed as follows: one cycle at 50°C for 2 min followed by 95°C for 20 s; amplification for 40 cycles at 95°C for 1 s followed by 60°C for 20 s; melting at 95°C for 1 s and 60°C for 20 s; dissociation at 95°C for 1 s. RefFinder^(34)^ was used to evaluate the stability of the reference genes (*GAPDH*, *RPOL2*, *EF1A* and *B2M*). The relative gene expression level was calculated using the ΔΔCt method^(35)^ using *GAPDH* as the reference gene for the proliferation genes and *B2M* as the reference gene for the genes related to differentiation (TaqMan probes).

**Table 4.** Primers used for qPCR (SYBR green, applied Biosystems)

Gene	Accession no.	Forward primer (5′–3 ′)	Reverse primer (5′–3′)	Amplicon sizes (bp)	Efficiency	R^2^
*GAPDH*	NM_002046·3	ATCCCATCACCATCTTCCAGGAGC	AAATGAGCCCCAGCCTTCTCCAT	120	102 %	0·98
*MKI67*	NM_002417·5	AAATTTGCTTCTGGCCTTCCC	TGTCACATTCAATACCCCTTCCA	185	97 %	0·99
*MYBL2*	NM_002466·4	CCAGCCACTTCCCTAACCG	CAGTGTCCACTGCTTTGTGC	151	100 %	0·99
*CCNB1*	NM_031966·4	CCTGCAGAAGAAGACCTGTGT	GACTGCTTGCTCTTCCTCAAGT	132	98 %	0·99
*PCNA*	NM_002592·2	CTTCCCTTACGCAAGTCTCAG	TTGAGTGCCTCCAACACCTT	188	110 %	0·99
*EF1A*	NM_001402·5	GACACGTAGATTCGGGCAAGTCCA	CCATCTCAGCAGCCTCCTTCTCAA	107	95 %	0·98
*RPOL2*	NM_000937·4	GCGCAATGAGCAGAACGGCG	ACTTCTGCATGGCACGGGGC	121	98 %	0·99

**Table 5.** Probes used for qPCR (TaqMan, Thermo Fisher)

Gene	Probe number
*RUNX2*	Hs00231692_m1
*ALP*	Hs00758162_m1
*BGLAP (OCN)*	Hs00609452_g1
*COL1a1*	Hs01028970_m1
*B2M*	Hs00187842_m1
*DKK1*	Hs00183740_m1
*AXIN2*	Hs00610344_m1

#### Microarray

Transcriptome analyses of vertebrae (six samples per group) were performed using 15 k Atlantic salmon DNA microarray SIQ-6 (GPL30031). RNA was extracted using a Biomek 4000 robot using Agencourt RNAdvance Tissue kit (Beckman Coulter), and the quality was assessed using an Agilent Bioanalyser 2100, RNA 6000 nano kit (RIN > 8). Microarrays, reagents, and equipment were manufactured by Agilent Technologies. RNA amplification and labelling were performed using the One-Colour Quick Amp Labelling Kit, and the Gene Expression Hybridisation kit was used for fragmentation of labelled RNA. After overnight hybridisation (17 h, 65°C, rotation speed of 0·01 g), the arrays were washed with Gene Expression Wash Buffers 1 and 2 and scanned using an Agilent scanner. Global normalisation was performed by equalising the mean intensities of all microarrays. The individual values for each feature were divided into the mean value of all samples to produce expression ratios (ERs). The log2-ER values were normalised with the locally weighted non-linear regression (Lowess). The difference from 0 to control was assessed by criteria: ER > 1·75-fold and *P* < 0·05.

### Fatty acid composition analysis

The method for analyses of total fatty acid composition, except VLC-PUFA, follows the same protocol as described in^(20)^. Briefly, total lipids were extracted using the Folch method^(36)^, and fatty acid methyl esters were prepared through trans-methylation with 2’,2’-dimethoxypropane, methanolic HCl, and benzene. Gas chromatography (GC) was utilised for the separation and analysis of methyl esters, employing an SGE BPX70 capillary column and a flame ionisation detector according to the method described by Lutfi *et al.*, 2023^(37)^.

For the VLC-PUFA, the same protocol for lipid extraction and methylation was used as explained above, but for VLC-PUFA in feeds and tissues, separate methods were used. For the VLC-PUFA-composition analysis of the diets, the protocol described in^(27)^ was employed, while for the VLC-PUFA composition of cells and whole-body extracts, a Scion TQ 8300 GC-MS/MS with a split/splitless injector (splitless 1 min) with a Restek Rxi-5ms capillary column (length, 30 m; internal diameter, 0·25 mm; and film thickness, 0·25 mM) was used. Hydrogen was used as the carrier gas, with split-injection and detector temperatures of 250°C and 270°C, respectively. The oven temperature started at 90°C for 1·5 min and was ramped up to 220°C at a rate of 30°C/min, then 275°C at a rate of 1·21°C/min and finally 310°C at a rate of 6·5°C/min. The instrument was run in multiple reaction monitoring (MRM) mode with 7·1–77·1 m/z and 79–55·1 m/z as transitions for VLC-PUFA and 143·1–55·1 m/z for C23:0^(38)^. The response factor for DHA (relative to C23:0) was calculated using standard solutions of known concentrations of DHA and C23:0. As no standards for VLC fatty acids were available for the VLC-PUFA, the same response factor as for DHA was assumed and used to calculate concentrations of VLC-PUFA.

### Cytokine quantitation

Cell culture medium from cells harvested for osteogenic differentiation markers was collected for cytokine analysis. Cytokine levels were measured using a Bio-Plex Pro™ Human Cytokine 8-plex assay (#171B5018M, Bio-Rad, Hercules, CA, USA) according to the manufacturer’s protocol.

### Statistical analysis

The assignment of feeds to each tank was randomised, and no animals were excluded from the study. All fish were included in the analysis. The tanks were coded and their identities unknown prior to data handling. Similarly, the cell culture experiments were conducted in a blinded manner until the data processing was completed. We employed one-way ANOVA to compare differences among the treatment groups. Significant differences (*P* < 0·05) were further analysed using Tukey’s honest significant difference (HSD) test for group ranking. Linear regression was applied where appropriate, to explore potential linear relationships between increasing levels of VLC-PUFA and the response variables. Statistical analyses were performed using JMP Pro (SAS Institute Inc., 1989–2019) and Microsoft Office Excel software. GraphPad Prism 9.3.9 was utilised for figure generation.

## Results

### Atlantic salmon *n*-3 VLC-PUFA feeding trial

#### Growth analysis

The mean body weights (BWs) of the fish at the start and end of the trial, along with the SGR and TGC, are presented in Table 6. The BW of the fish increased threefold during the trial, from a mean start weight of 5·7 g to a mean final weight of 17 g. There were no significant differences in final BW (*P* = 0·28), SGR (*P* = 0·55) or TGC (and *P* = 0·55) between the different diet groups. There were no observed mortalities in any of the dietary groups.

**Table 6.** Mean body weights and growth rates of salmon fed the experimental diets

	Diet groups										
	Ctr		L-VLC		I-VLC		MH-VLC		H-VLC		
	Mean	sem	Mean	sem	Mean	sem	Mean	sem	Mean	sem	*P*-value
BW (g) start	5·71	0·01	5·75	0·02	5·72	0·01	5·76	0·01	5·73	0·02	0·24
BW (g) end	17·53	0·03	17·33	0·03	17·23	0·15	17·1	0·20	17·13	0·20	0·28
SGR	3·13	0·33	3·06	0·04	3·05	0·03	3·05	0·05	3·03	0·06	0·55
TGC	1·73	0·02	1·69	0·02	1·69	0·02	1·68	0·03	1·67	0·04	0·55

Ctr, Control; L-VLC, Low-VLC; I-VLC, Intermediate-VLC; MH-VLC, Medium-High-VLC; H-VLC, High-VLC; BW, body weight; sem, standard error of the mean; SGR, specific growth rate; TGC, thermal growth coefficient.

*n* 3 tanks, 100 fish per tank per diet group.

There were no significant differences in bioavailability of the minerals phosphorus (P) or magnesium (Mg) between the different diet groups (Table 7). However, zinc (Zn) showed a significant difference between the groups, with a moderately lower bioavailability in the I-VLC and H-VLC diet groups compared with the control group (*P* = 0·01).

**Table 7.** Bioavailability of minerals (% of bioavailable minerals from the diet) in Atlantic salmon

	Diet groups						
	Ctr		I-VLC		H-VLC		
	Mean	sem	Mean	sem	Mean	sem	*P*-value
P	41·78	1·99	42·95	0·42	39·50	0·80	0·23
Mg	39·11	2·31	38·10	1·07	37·55	2·67	0·87
Zn	27·88^a^	1·29	21·72^b^	0·47	21·11^b^	1·31	0·01
DM	15·10	0·21	14·20	0·23	13·70	0·38	0·04

Data are presented as mean (standard error of mean) (sem).

Ctr, Control; I-VLC, Intermediate-VLC; H-VLC, High-VLC; P, phosphorus; Mg, magnesium; Zn, Zinc; (% DM in faeces).

The letters a and b denote significant differences between the groups.

#### Whole-Body fatty acid composition of Atlantic salmon

Analysis of whole-body fatty acid composition showed there were no significant differences in the amount of fatty acids containing ≤ 22 carbon atoms between the different dietary groups (Table 8). The aim of obtaining the same whole-body content of EPA and DHA in all dietary groups was achieved, with a body content of 14 mg/g of these fatty acids.

**Table 8.** Fatty acid composition of whole-body homogenate of Atlantic salmon

	Diet groups										
	Ctr		L-VLC		I-VLC		MH-VLC		H-VLC		
	Mean	sem	Mean	sem	Mean	sem	Mean	sem	Mean	sem	*P*-value (one-way ANOVA)
14:0	4·96	0·32	4·68	0·34	4·47	0·27	4·91	0·34	4·86	0·18	0·7684
15:0	0·27	0·09	0·36	0·02	0·34	0·02	0·37	0·02	0·37	0·01	0·4934
16:0	12·59	0·81	13·03	0·81	12·73	0·61	13·80	0·89	13·63	0·36	0·7032
17:0	0·19	0·02	0·18	0·01	0·18	0·02	0·18	0·02	0·25	0·05	0·2395
18:0	2·03	0·40	2·38	0·15	2·32	0·07	2·46	0·18	2·62	0·10	0·4404
Sum of SFAs	20·36	1·17	20·90	1·35	20·31	1·00	21·94	1·39	21·97	0·61	0·7272
16:1 *n*-7	4·24	0·25	4·26	0·30	4·32	0·37	4·67	0·37	4·42	0·19	0·8404
18:1 *n*-9	13·19	1·50	14·63	1·31	13·56	0·88	14·70	1·34	15·14	0·65	0·7411
20:1 *n*-7	9·41	0·39	9·31	0·59	8·70	0·54	9·74	0·58	9·57	0·37	0·6642
20:1 *n*-11	1·75	0·15	1·57	0·09	1·53	0·05	1·66	0·09	1·64	0·06	0·5538
22:1 *n*-9	1·17	0·24	0·98	0·05	0·82	0·06	1·09	0·17	0·91	0·05	0·4411
22:1 *n*-11	12·03	1·27	10·63	0·60	10·40	0·43	11·21	0·54	11·10	0·29	0·5645
24:1 *n*-9	0·68	0·29	0·42	0·26	0·42	0·25	0·73	0·29	0·70	0·26	0·8488
Sum of MUFAs	46·37	2·69	45·27	4·83	44·13	4·38	48·21	5·25	47·81	2·50	0·7200
18:2 *n*-6	3·86	0·16	3·81	0·17	3·89	0·03	4·09	0·11	4·03	0·04	0·4388
18:3 *n*-6	0·15	0·02	0·15	0·01	0·17	0·00	0·16	0·02	0·17	0·00	0·9034
20:2 *n*-6	0·33	0·07	0·40	0·02	0·39	0·01	0·42	0·02	0·43	0·01	0·3725
20:3 *n*-6	0·17	0·01	0·18	0·02	0·16	0·01	0·18	0·02	0·17	0·00	0·8469
20:4 *n*-6	0·28	0·01	0·28	0·01	0·28	0·01	0·29	0·01	0·29	0·00	0·9545
22:4 *n*-6	0·25	0·04	0·31	0·02	0·28	0·01	0·28	0·05	0·32	0·01	0·6263
Sum of *n*-6 PUFA	4·95	0·17	5·13	0·16	5·08	0·19	5·34	0·27	5·44	0·09	0·3952
18:3 *n*-3	1·02	0·02	0·97	0·05	0·97	0·01	1·03	0·05	1·01	0·02	0·5780
20:4 *n*-3	0·55	0·10	0·62	0·04	0·54	0·09	0·60	0·06	0·57	0·11	0·9570
20:3 *n*-3	0·13	0·00	0·12	0·01	0·12	0·00	0·12	0·00	0·13	0·00	0·8376
20:5 *n*-3	3·56	0·41	2·95	0·10	3·10	0·01	2·97	0·15	3·18	0·11	0·2803
22:5 *n*-3	1·25	0·22	0·93	0·03	1·15	0·16	1·31	0·21	1·31	0·19	0·5454
22:6 *n*-3	10·04	1·14	11·19	0·40	11·15	0·20	11·68	0·41	11·74	0·30	0·3315
Sum of *n*-3 PUFA	16·75	1·01	17·00	0·60	17·06	0·50	17·90	0·46	18·16	0·30	0·4598
Sum EPA/DHA	13·61	0·74	14·14	0·50	14·25	0·20	14·65	0·39	14·92	0·37	0·3952
Sum others^*^	4·45	0·28	3·99	0·56	4·55	0·19	4·92	0·47	4·99	0·14	0·3684
SUM FA	91·80	3·35	93·26	4·73	91·81	3·06	97·52	5·39	97·81	2·74	0·6918

Data are presented as mg/g tissue.

Ctr, Control; L-VLC, Low-VLC; I-VLC, Intermediate-VLC; MH-VLC, Medium-High-VLC; H-VLC, High-VLC.

* Others include 20:0, 24:0, 14:1 *n*-5, 15:1, 16:1 *n*-5, 17:1 *n*-7, 18:1 *n*-7, 19:1, 22:1 *n*-7, 16:2 *n*-6, 16:2 *n*-3.

**Figure 1.**
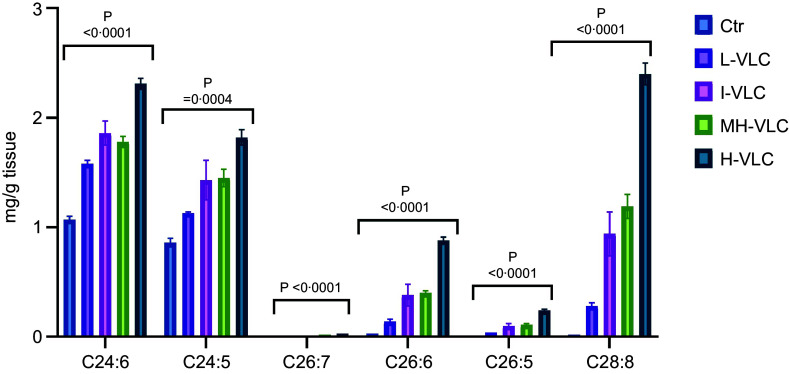
VLC-PUFA composition of whole-body homogenate. Ctr, Control; L-VLC, Low-VLC; I-VLC, Intermediate-VLC; MH-VLC, Medium-High-VLC; H-VLC, High-VLC.

The VLC-PUFA composition of whole-body homogenate showed a significant increase in VLC-PUFA from the diet, with the highest amount in the H-VLC diet group (Table 9, Fig. 1). The 24:6 *n*-3 content increased from 1·07 mg/g tissue in the L-VLC diet group to 2·31 mg/g tissue in the H-VLC diet group (*P* < 0·0001), and 24:5 *n*-3 increased from 0·86 mg/g tissue in the L-VLC diet group to 1·82 mg/g tissue in the H-VLC diet group (*P* = 0·0004). While 26:7 *n*-3 and 26:5 *n*-3 were both undetected in the L-VLC diet group, they were detected at levels of 0·03 mg/g tissue and 0·24 mg/g tissue, respectively, in the H-VLC diet group (*P* < 0·0001). Furthermore, 26:6 *n*-3 increased from 0·03 mg/g tissue in the L-VLC diet group to 0·88 mg/g tissue in the H-VLC diet group (*P* < 0·0001) and 28:8 *n*-3 increased from 0·02 mg/g tissue in the L-VLC diet group to 2·40 mg/g tissue in the H-VLC diet group (*P* < 0·0001).

**Table 9.** Mineral composition of Atlantic salmon skin

	Ctr		L-VLC		I-VLC		MH-VLC		H-VLC		
	Mean	sem	Mean	sem	Mean	sem	Mean	sem	Mean	sem	*P*-value
P	10 133	867	11 000	577	11 667	333	10 667	333	13 000	577	0·02*
Ca	15 333	1667	17 000	1155	17 667	882	17 333	667	20 333	882	0·01*
Mg	453	33	467	17	490	15	487	23	540	32	0·02*
Zn	47	6	51	1	51	2	49	4	56	6	0·21

Ctr, Control; L-VLC, Low-VLC; I-VLC, Intermediate-VLC; MH-VLC, Medium-High-VLC; H-VLC, High-VLC; P, phosphorus; Ca, calcium; Mg, magnesium; Zn, zinc.

Data are presented as mg/kg. *P*-value is of linear regression analysis.

* Indicates significant difference (*P* < 0·05).

#### Skeletal morphology and mineral component analysis of skin and vertebrae

The results of the mineral component analysis of salmon skin and vertebrae are presented in Tables 9 and 10, respectively. In the skin from Atlantic salmon fed increasing dietary levels of VLC-PUFA, P increased by ∼28 % in the High-VLC-PUFA group compared with the control group, and calcium (Ca) and Mg increased by ∼32 % (*P* = 0·01) and ∼19 % (*P* = 0·02), respectively.

**Table 10.** Mineral composition of Atlantic salmon vertebrae

	Ctr		L-VLC		I-VLC		MH-VLC		H-VLC		
	Mean	sem	Mean	sem	Mean	sem	Mean	sem	Mean	sem	*P*-value
P	18 000	0	18 667	1333	18 333	882	19 000	1528	22 000	0	0·02*
Ca	32 333	667	33 000	3000	32 667	1667	34 333	32 823	40 333	333	0·02*
Mg	603	7	607	30	620	15	627	20	690	6	0·01*
Zn	140	26	127	15	140	12	123	13	173	26	0·29

Ctr, Control; L-VLC, Low-VLC; I-VLC, Intermediate-VLC; MH-VLC, Medium-High-VLC; H-VLC, High-VLC; P, phosphorus; Ca, calcium; Mg, magnesium; Zn, zinc.

Data are presented as mg/kg. *P*-value is of linear regression analysis.

* Indicates significant difference (*P* < 0·05).

In the vertebrae from Atlantic salmon fed increasing dietary levels of VLC-PUFA, P increased by 22 % from the control group to the High-VLC-PUFA group (*P* = 0·02). Ca and Mg also increased with increasing levels of VLC-PUFA in the feed; Ca increased by ∼25 % (*P* = 0·02) and Mg increased by around 14 % (*P* = 0·01).

### Radiography of Atlantic salmon vertebrae

**Figure 2.**
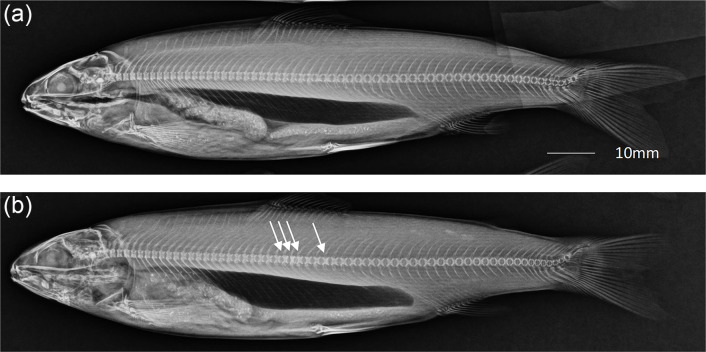
Fish sampled at the end of the experiment. (a) Fish with normal skeletal structures. (b) Fish with hyper-dense vertebrae under the dorsal fin (arrows). Affected vertebrae displayed a higher radiodensity than adjacent vertebrae, with some minor changes in shape.

X-ray analyses of the fish in the different diet groups showed no significant differences in pathology, and the skeletal structures appeared uniform and well-developed. Hyper-dense vertebrae^(39)^ were observed in five of the 75 fish examined; however, these were randomly distributed between the diet groups (Fig. 2). The hyper-dense lesions were similar in all affected individuals and consisted of one to three vertebrae with increased radiodensity compared with the adjacent structures. The lesions were localised to the cranium and under the dorsal fin.

### CT analyses

**Figure 3.**
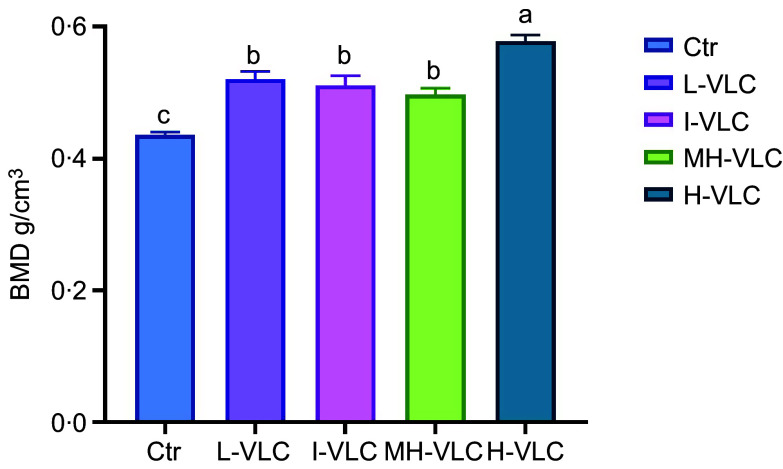
CT analysis of Atlantic salmon (*n* 10–15 fish per diet group). Ctr, Control; L-VLC, Low-VLC; I-VLC, Intermediate-VLC; MH-VLC, Medium-High-VLC; H-VLC, High-VLC. Significance was set at *P* < 0·05. The letters a–c denotes significant differences between the groups.

CT analyses of fish from the different diet groups revealed a significantly higher BMD in the H-VLC-diet group compared with the other diet groups, with the lowest levels observed in the control group (Fig. 3).

#### Microarray analysis results

**Figure 4.**
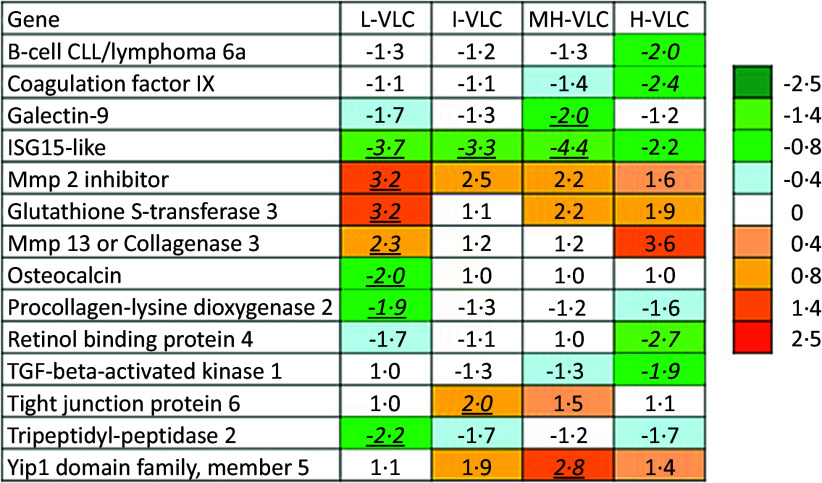
Genes with known or possible roles in bone development. The heatmap presents fold changes compared with the control. Differential expressions are indicated by underlined bold italics. L-VLC, Low-VLC; I-VLC, Intermediate-VLC; MH-VLC, Medium-High-VLC; H-VLC, High-VLC.

The dietary effects on gene expression in bone were small, with two to 10 differentially expressed genes identified. Only two genes showed differences in more than one group. Several differentially expressed genes may be associated with bone-related processes (Fig. 4). *B-cell lymphoma 6a* (*Bcl6*) was downregulated twofold in the H-VLC diet group, and *coagulation factor IX* was also downregulated in the same group. *Galectin-9* showed reduced expression in the MH-VLC diet group, while *isg15* was significantly downregulated in three dietary groups. *Mmp2 inhibitor* showed the highest expression in the L-VLC diet group, and c*ollagenase 3* (*mmp13*) showed significant up-regulation in both the L-VLC and High-VLC diet groups. *Osteocalcin* was downregulated in the L-VLC diet group, and the same was *procollagen-lysine dioxygenase 2*. Finally, *retinol-binding protein 4* was downregulated in the MH-VLC diet group.

### *In vitro* cell culture of hFOB 1·19 human foetal osteoblasts

#### Fatty acid composition of human foetal osteoblasts

The cell culture media were supplemented with 2 µM VLC-PUFA, and a 2 µM DHA group was included as positive control, as well as a control group without any supplementation. The fatty acid composition of the cells after 7 d of supplementation is presented in Table 11. Levels of EPA (20:5 *n*-3) were significantly different between the three groups, with a slightly higher level in the 2 µM VLC-PUFA group, although the supplemented VLC-PUFA concentrate did not contain this fatty acid (*P* = 0·0232). DHA (22:6 *n*-3) was also significantly different between the groups, with the highest level observed in the 2 µM DHA group (*P* = 0·0013), likely due to the VLC-PUFA concentrate containing this fatty acid. There was also a significant difference in the sum of *n*-3 PUFA, with the highest level in the 2 µM VLC-PUFA group (*P* = 0·0248). Levels of the VLC-PUFA 26:6 *n*-3 were significantly higher in the group supplemented with VLC-PUFA concentrate (2 µM) compared with the control and DHA groups (*P* = 0·0389). The same was true also for 26:5 *n*-3 (*P* = 0·304) and 28:8 *n*-3 (*P* = 0·0022).

**Table 11.** Fatty acid composition of hFOB cells

	Control		2 µM DHA		2 µM VLC-PUFA		
	Mean	sem	Mean	sem	Mean	sem	*P*-value (one-way ANOVA)
14:0	37·3	3·02	38·3	2·23	41·8	1·79	0·4331
16:0	642·8	4·6	648·5	40·4	626·7	22·2	0·8401
17:0	13·4	0·3	12·5	0·7	12·5	0·5	0·4179
18:0	316·7	8·1	315·8	20·2	304·2	7·7	0·7698
20:0	11·1	0·2	10·1	0·6	10·1	0·4	0·2270
22:0	8·3	0·1	7·3	0·8	8·3	0·3	0·2975
Sum of SFAs	1040·6	0·9	1045·0	58·6	1019·8	32·3	0·8878
18:1 *n*-7	141·0	2·4	135·0	5·8	134·6	3·1	0·4985
18:1 *n*-9	573·7	10·5	554·6	19·9	546·5	14·1	0·4789
20:1 *n*-9	19·3	0·4	16·2	2·6	18·3	0·4	0·3994
20:1 *n*-11	5·8	0·4	7·1	1·1	7·6	1·5	0·4990
24:1 *n*-9	3·9	2·0	3·9	2·0	5·9	0·2	0·6398
Sum of MUFAs	907·7	12·7	871·5	30·9	831·9	45·2	0·3255
16:2 *n*-6	47·7	10·4	52·5	3·0	49·1	3·7	0·8640
18:2 *n*-6	41·9	0·8	40·6	1·5	41·4	1·2	0·7394
18:3 *n*-6	12·4	0·7	11·5	1·1	11·9	1·1	0·8032
20:2 *n*-6	19·0	0·3	18·8	0·8	17·3	0·5	0·1299
20:3 *n*-6	21·9	0·2	20·6	0·9	22·0	0·6	0·2916
20:4 *n*-6	78·8	1·3	106·7	30·3	76·9	2·0	0·4535
22:4 *n*-6	13·7	0·3	13·7	0·5	14·4	0·5	0·4756
Sum of *n*-6 PUFA	197·6	18·0	226·2	42·3	211·0	7·7	0·7633
20:4 *n*-3	9·1	0·3	8·8	1·3	7·1	0·5	0·2897
20:5 *n*-3	18·7^a^	0·4	19·3^ab^	0·7	21·7^b^	0·6	0·0232^*^
22:5 *n*-3	27·2	0·4	26·2	0·8	26·9	1·5	0·8063
22:6 *n*-3	75·5^a^	1·0	96·3^c^	2·7	84·8^b^	2·1	0·0013^*^
Sum of *n*-3 PUFA	142·3^a^	3·3	164·0^ab^	5·1	173·6^b^	8·3	0·0248^*^
SUM EPA/DHA	94·2^a^	1·4	115·6^b^	3·4	106·5^b^	2·7	0·0036^*^
24:6 *n*-3	49·3	4·2	56·5	1·8	62·2	7·1	0·2466
24:5 *n*-3	67·4	4·1	76·5	3·7	84·5	11·3	0·3161
26:6 *n*-3	2·6^a^	0·2	3·3^ab^	0·3	6·7^b^	1·5	0·0389^*^
26:5 *n*-3	1·1^a^	0·2	1·7^ab^	0·5	4·6^b^	1·2	0·0304^*^
28:8 *n*-3	0·0^a^	0·0	0·0^a^	0·0	11·8^b^	2·6	0·0022^*^
SUM FA	2635·3	55·8	2670·3	63·7	2672·8	73·6	0·9021
Sum others^†^	180·4	3·9	168·3	25·8	186·7	10·1	0·7292

Data are presented as µg/g sample (*n* 3).

^a,b^Mean values within each column with different superscript letters are significantly different.

* Indicates significant difference (*P* < 0·05).

† Included in others 15:0, 15:1, 16:1 *n*-7, 16:1 *n*-5, 17:1 *n*-7, 18:1 *n*-11, 20:1 *n*-7, 16:2 *n*-3, 18:4 *n*-3.

#### qPCR

The different diet groups showed no significant differences in the expression of the proliferation markers: marker of proliferation Ki-67 (*MKI67*), MYB proto-oncogene like 2 (*MYBL2*), cyclin B1 (*CCNB1*), and proliferating cell nuclear antigen. However, there was a trend towards higher expression of proliferating nuclear antigen in the control group on day 2, which was reversed on day 3, with a trend towards higher expression in the 1 µM VLC-PUFA group and 2 µM DHA group, albeit NS (data presented in online Supplementary Fig. S1).

**Figure 5.**
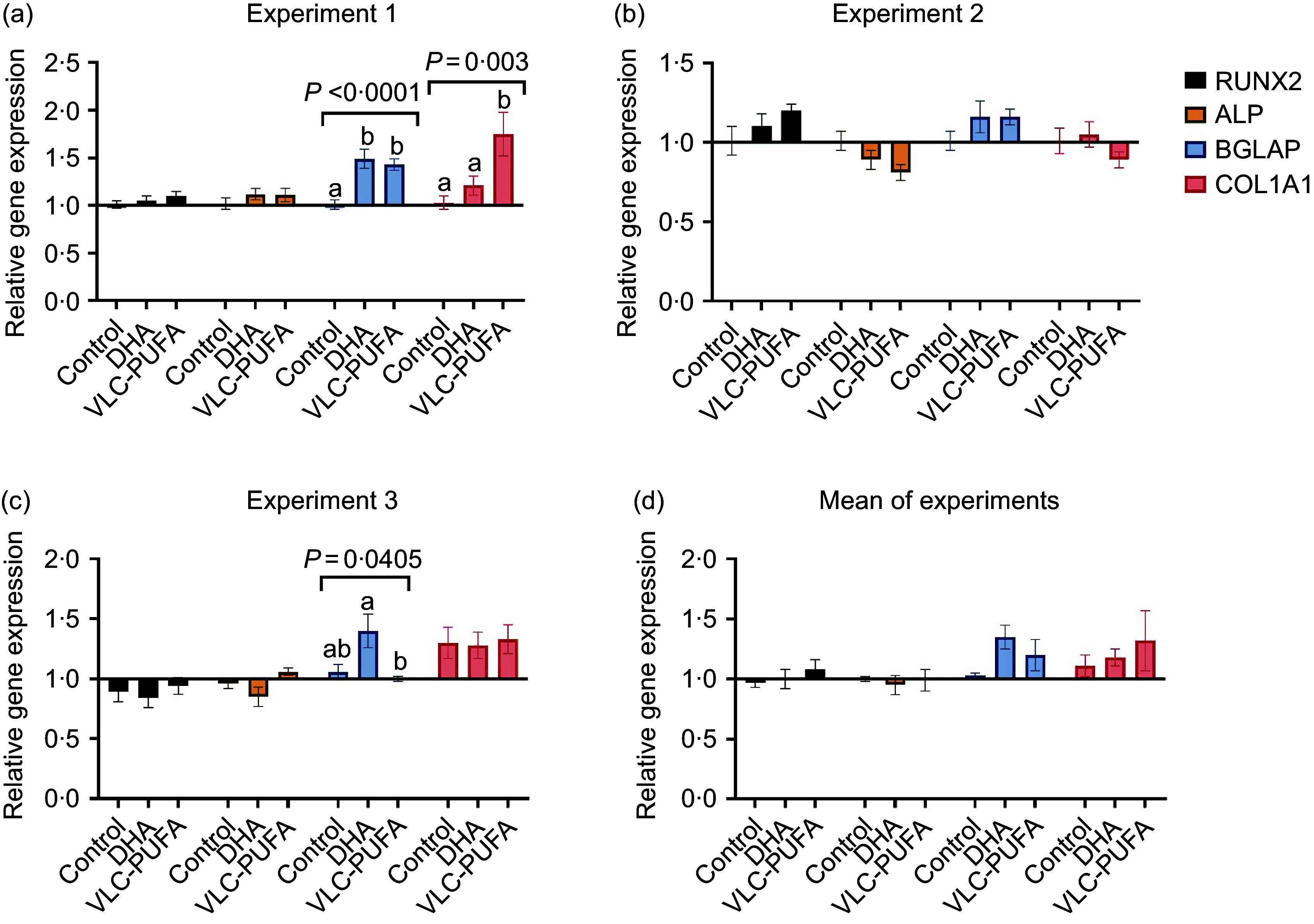
Gene expression of the differentiation markers RUNX2, ALP, BGLAP, and COL1A1 in response to supplementation of VLC-PUFA or DHA. hFOB 1·19 cells were supplemented with VLC-PUFA or DHA (1 and 2 µM of each, doses are presented combined). Total RNA was extracted and qPCR analysis for gene expression was performed. (a) Experiment 1. (b) Experiment 2. (c) Experiment 3. (d) Mean of experiments. Data are presented as mean (s em). Comparisons were conducted using one-way ANOVA and Tukey HSD test. The significance level was set to *P* < 0·05.

As there were no significant differences between the two different test doses of the supplemented fatty acids, the combined effect of 1 and 2 µM VLC-PUFA as well as 1 and 2 µM DHA are presented for the cells cultured for analysis of the differentiation markers: *RUNX2*, *ALP*, *BGLAP* and *COL1A1* (Fig. 5). There were significantly higher levels of *BGLAP* and *COL1A1* in both the DHA and VLC-PUFA groups compared with the control group in experiment 1 (Fig. 5(a)). However, when combining the means of all three experiments, there were no significant differences, although there was a slight trend of increased levels of *BGLAP* and *COL1A1* in the DHA and VLC-PUFA supplemented groups (Fig. 5(d)).

#### Cytokine quantitation

**Figure 6.**
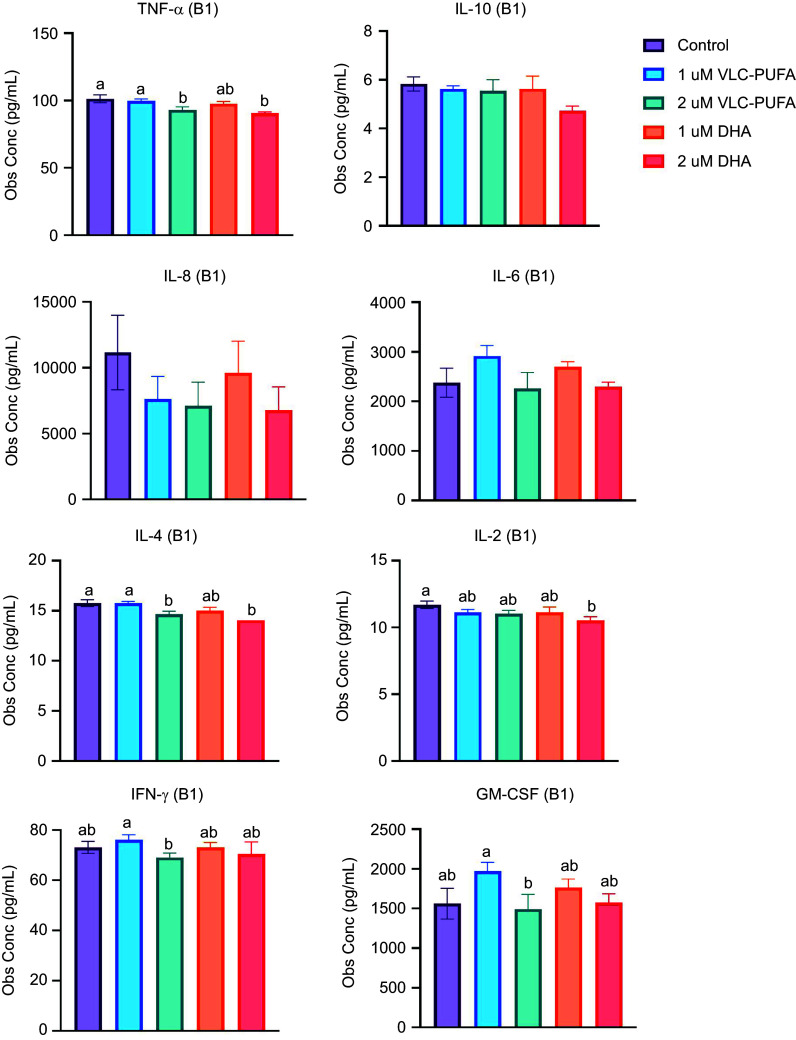
Cytokine expression of growth medium from hFOB 1·19 cells cultured for gene expression analysis of differentiation markers. Data are presented as mean (sem) (2–6 replicates in batch 1 (B1)).

Cytokine quantitation of growth medium from hFOB 1·19 cells cultured for gene expression analysis of differentiation markers revealed significant differences in the expression of TNF-*α*, IL-4, IL-2, IFN-*γ* and GM-CSF, with lower expression in the 2 µM VLC-PUFA group. Expression of GM-CSF was significantly higher in the 1 µM VLC-PUFA group, while it was lowest in the 2 µM VLC-PUFA group (Fig. 6). Although not statistically significant, there was a slight trend towards lower expression of IL-8 in the two VLC-PUFA and 2 µM DHA groups compared with the control.

## Discussion

The present study investigated the impact of dietary *n*-3 VLC-PUFA on skin and bone mineral composition, BMD, and gene expression profiles within the skeletal systems of Atlantic salmon. The choice of Atlantic salmon as an *in vivo* model was based on several considerations, in particular the fact that several major physiological and morphological features are shared between teleost and mammalian bones^(21)^. The physiological and genetic proximity of fish, in this case, Atlantic salmon, to humans allows for insightful extrapolation of findings relevant to our own species^(40)^, and the feasibility of controlled dietary interventions and their relatively rapid growth facilitates a comprehensive assessment of the dietary impact during the developmental period^(41,42)^. To bridge the gap between animal models and the potential relevance for human skeletal health, we also investigated the effects on human foetal osteoblasts *in vitro* to elucidate the translational relevance of our findings.

The Atlantic salmon feeding trial demonstrated a substantial threefold increase in BW of the fish, indicating successful growth during the experimental period. The SGR and TGC values among the different dietary groups showed that supplementation of VLC-PUFA did not exert significant effects on the overall growth performance of the fish, and X-ray analysis also showed no notable effects on the skeletal morphology. However, the fish in all the dietary groups had vertebrae mineral contents within the considered normal range^(43)^, which is expected since all the fish were fed minerals according to their requirements. This likely explains why no major pathologies or deviations from normal morphology of vertebrae were recorded with X-ray analyses. The good growth and survival (100 %) of the experimental fish fed increasing dietary levels of VLC-PUFA confirmed that there were no major fish health concerns regarding this ingredient. These results align with our previous studies, albeit with variations in VLC-PUFA composition and dietary concentrations^(20,27)^, and are consistent with findings demonstrating the safety of VLC-FAs^(44)^. The whole-body fatty acid composition of the salmon revealed a linear increase in several VLC-PUFA, including 24:6 *n*-3, 24:5 *n*-3, 26:7 *n*-3, 26:5 *n*-3, 26:6 *n*-3 and 28:8 *n*-3. There were no significant differences in EPA and DHA, in accordance with the content of these fatty acids in the diet. These findings demonstrate the successful deposition of VLC-PUFA in fish through dietary supplementation, as also previously shown^(20,27)^. Other studies have demonstrated that VLC-PUFA are present in sphingolipids in skin and sperm^(45)^ and predominantly in total phospholipids in neuronal tissue and the brain^(46,47),^ while we have previously shown in^(27)^ that VLC-PUFA are also incorporated into TAG which the bone marrow is very rich in^(48)^. In our study, we focused on total lipids including all polar and unpolar lipids in the whole body, as it remains unknown which lipid classes are most important for VLC-PUFA in tissues such as bone. This will be an important area for further investigation.

A significant correlation between increased dietary *n*-3 VLC-PUFA and elevated P, Ca, and Mg content within both the skin and vertebrae was also found, as well as increased BMD of salmon vertebrae. Similar findings were shown in a study where the effects of long-term dietary fish oil supplementation on bone health were investigated^(49)^. This study demonstrated that fish oil supplementation significantly increased tibial mineral content, mechanical properties and histological parameters compared with other dietary fats in Japanese quail^(49)^. As the sum of EPA and DHA levels was kept constant between all dietary groups in our study, our findings suggest an independent effect of the VLC-PUFA concentrate. However, it is important to address whether the observed differences were a direct consequence of VLC-PUFA intake or whether other factors, such as altered mineral absorption across the intestinal barrier, could contribute to the higher levels. Additionally, while our study focused on P, Ca and Mg, consideration of other trace minerals like copper, manganese and selenium, which also contribute to bone mineralisation, could provide valuable insights in future research.

To investigate the possible role of intestinal absorption in the elevated mineral content, we assessed the intestinal bioavailability of the dietary minerals P, Mg and Zn across the different dietary groups. No differences in the intestinal bioavailability of P and Mg between the dietary groups suggest that the increased mineral content in the skin and vertebrae is attributed to other functions than intestinal mineral absorption. The only mineral that showed significant differences was Zn, which was lower in the VLC-PUFA-supplemented groups. This was however not reflected in the vertebrae and skin samples, indicating that the elevated mineral levels observed in the tissues are unlikely to be driven by differences in intestinal uptake alone. This is an interesting finding, as prior studies have shown the potential of *n*-3 LC-PUFA supplementation to enhance bone calcium content through increased intestinal absorption and decreased urinary excretion^(50)^. P and Ca are known to be the most relevant nutrients to bone health, as they comprise around 80–90 % of the mineral content of bone hydroxyapatite^(51)^, while Mg depletion has been shown to have a profound effect on bone, characterised by impaired bone growth, reduced osteoblast number and increased osteoclast number^(52)^. Previous studies have also demonstrated that increasing dietary levels of fish oil in Atlantic salmon feed improves the mineral content of the fish^(53)^. Our observed correlation between *n*-3 VLC-PUFA and mineral content further underscores the possibility that VLC-PUFA could play a pivotal role in shaping the mineral composition of fish tissues, potentially influencing overall skeletal health. While vitamin D in fish oil is known to contribute to calcium absorption and bone health^(54)^, the natural vitamin D content in the raw fish oil is largely depleted during the refining process^(55)^. Therefore, our VLC-PUFA concentrate likely contains minimal levels of vitamin D, suggesting that its influence on the observed effects is minimal, and making VLC-PUFA key factors in these outcomes.

Our study, as well as evidence from both human and animal studies, highlights the intricate link between dietary lipids and bone metabolism^(50)^. Notably, *n*-3 fatty acids have garnered considerable attention due to their impact on enhancing BMD^(56–58)^ and positive influence on bone metabolism^(50,59)^. Recent studies have identified strong association between gene variants of ELOVL with bone density, with evidence linking these variants to the incidence of fractures^(60)^. Furthermore, microRNA regulation of ELOVL4 has been shown to be implicated in fracture susceptibility, as demonstrated by Dai *et al.* ^(61)^. Additionally, a study by Arsic *et al.* ^(62)^ has shown that reduced PUFA levels in plasma may influence hip fracture repair and contribute to femoral fracture susceptibility in the elderly, underscoring the potential benefit of PUFA intervention in both fracture prevention and healing. In our study, analysing gene expression in the vertebrae revealed subtle yet significant impacts on bone-related processes. Although the number of differentially expressed genes was relatively modest, their potential implications for bone health are noteworthy. In particular, the observed twofold down-regulation of *Bcl6* in the H-VLC diet group underscores the potential influence of *n*-3 VLC-PUFA on regulatory pathways in bone, since *Bcl6* is recognised for its pivotal role in orchestrating osteoblast activation and inhibition^(63,64)^. *Coagulation factor IX*, which is known to be essential for proper skeletal development^(65)^, was downregulated in the vertebrae of the H-VLC diet group. Furthermore, *galectin-9*, which is implicated in osteoblast differentiation^(66)^, displayed reduced expression in the MH-VLC diet group. These regulations point to potential intricacies in the interaction between dietary factors and molecular mechanisms governing bone cell differentiation. Conversely, *mmp2 inhibitor*, which is associated with enhanced bone formation and prevention of bone loss^(67)^, exhibited the highest expression in the L-VLC diet group. Additionally, *collagenase 3* (*mmp13*), which is critical for restructuring the collagen matrix during bone mineralisation, showed significant up-regulation in both the L-VLC and H-VLC diet groups. This dynamic expression pattern further hints at complex regulatory mechanisms influenced by dietary factors. Notably, some genes critical for bone formation showed contradictory expression patterns. *Osteocalcin*, an essential factor for bone formation^(68)^, and *procollagen-lysine dioxygenase 2*, which plays a role in forming robust collagen fibres^(69)^, were downregulated in the L-VLC diet group and the higher VLC-PUFA diet groups were not significantly affected. Moreover, *retinol-binding protein 4*, linked to BMD in conditions such as type 2 diabetes and osteoporosis^(70)^, emerged as a downregulated gene in the MH-VLC diet group.

Genotoxicity studies have established that VLC-FAs do not exhibit genotoxic potential at doses up to 1200 mg/kg BW per day (using a fish oil concentrate where VLC-PUFA accounted for 10 % of the fatty acids), as reported in Sprague Dawley rats over a 90-day feeding period^(71)^. In our study on Atlantic salmon, the fish were administered feeding containing various concentrations of VLC-PUFA concentrate, with the highest doses being 1·9 % VLC-PUFA concentrate per 100 g of feed. The fish consumed an estimated amount of VLC-PUFA concentrate that equates to 380 mg/kg BW per day. This intake level is well below the established NOAEL of 1200 mg/kg BW per day, which is consistent with the no adverse effects observed in the fish in our trial. Nevertheless, the highest dosage provided in our study, which equates to 380 mg/kg BW per day, demonstrated a significant increase in BMD, which may represent a relevant safe dietary dose also for humans based on the rat toxicity trial. This suggests a potential positive biological response that may be relevant for further exploration in human studies.

In contrast to our *in vivo* study, where we balanced EPA and DHA levels in the tissues to isolate the effects of VLC-PUFA, the aim of our *in vitro* study was to directly compare the effects of VLC-PUFA against those of DHA. By doing so, we seek to clarify the distinct roles and potential advantages of VLC-PUFA in cellular processes, relative to DHA, within a controlled environment. Our *in vitro* studies with human foetal osteoblast cells supplemented with VLC-PUFA or DHA identified trends in the expression of key markers associated with bone development and maturation, namely *BGLAP* and *COL1A1* ^(72)^, in both the DHA and VLC-PUFA groups compared with the control. Although not statistically significant, these trends aligned with *in vivo* findings, suggesting the possibility of DHA and VLC-PUFA influencing the expression of key markers associated with bone development and maturation. However, further investigations are needed to explore the underlying mechanisms and confirm these trends. As several inflammatory cytokines are known to be involved in bone metabolism^(73)^, we also analysed cytokine expression in the growth medium from the human foetal osteoblast cells analysed for expression of bone differentiation markers. There was slightly lower level of IL-8 in the VLC-PUFA-supplemented groups and slightly lower level of IL-6 in the 2 µM VLC-PUFA group. Additionally, TNF-*α*, IL-4, IFN-*γ* and GM-CSF were significantly lower in the 2 µM VLC-PUFA group compared with control. These trends could indicate a subtle modulation of cytokine responses due to VLC-PUFA supplementation. As TNF-*α* is known to play a dual role in bone remodelling through stimulation of osteoclastogenesis and inhibition of osteoblast function^(74)^, reduced levels of TNF-*α* can help maintain a balance between bone resorption and formation. Furthermore, IL-4 and IFN-*γ* have inhibitory effects on bone resorption^(75)^, and reduced levels may thus also contribute to maintaining a balance between bone resorption and formation.

The impacts of *n*-3 VLC-PUFA on bone formation were studied in both Atlantic salmon and a human cell line. While notable effects were observed in both cases, the differences between the two methods preclude direct comparison. For one, we acknowledge that the dietary oil used in the fish model contained a mix of VLC-PUFA and shorter chain fatty acids including EPA and DHA, while a more pure VLC-PUFA concentrate was used in the human cell model. These differences may affect the interpretation of the functions of VLC-PUFA between the two models. For instance, it is previously shown that EPA and DHA from diet may to a moderate degree be further converted to certain VLC-PUFA^(27)^. It is therefore essential to acknowledge the potential variations between an *in vivo* fish model and a human *i*n vitro cell model, both in their response to dietary fatty acids, as well as their physiological differences. The observed similarities between *in vivo* findings from fish and *in vitro* findings from human cells are still interesting pointing to the possible important roles of VLC-PUFA in bone development in vertebrates across different species.

### Conclusions

Our study provides initial evidence that dietary supplementation with *n*-3 VLC-PUFA may increase the mineral content of the skin and vertebrae of Atlantic salmon and affect the expression of several genes involved in bone-related processes. CT analyses further demonstrated a significant increase in BMD with increasing *n*-3 VLC-PUFA in the diet, consistent with the findings of increased mineral content of the vertebrae. The human foetal osteoblast experiments showed trends towards increased expression of the genes *BGLAP* and *COL1A1* which are known to have important functions during bone development. The presented study provides novel insight into the intricate relationship between dietary *n*-3 VLC-PUFA, bone and skin mineral content, which to our knowledge has not previously been explored. Overall, our study contributes valuable insight into the potential role of *n*-3 VLC-PUFA in bone health. Further studies into the underlying molecular mechanisms are needed to elucidate the complex relationship between nutrition and bone physiology, particularly in relation to *n*-3 VLC-PUFA, and to conclude if VLC-PUFA have different functions in fish and mammals.

## Supporting information

Torrissen et al. supplementary material Torrissen et al. supplementary material

## Supplementary material

For supplementary material/s referred to in this article, please visit https://doi.org/10.1017/S0007114524003052
