# Supplementary material for: Effects of n-3 very-long-chain PUFA on bone mineralisation
Source: Br J Nutr. 2024 Nov 29;133(2):145–60. doi: 10.1017/S0007114524003052 (PMC11813626; doi:10.1017/S0007114524003052)
Supplement: Torrissen et al. supplementary material [file S0007114524003052sup001.docx]

**Supplementary Material**

Figure S1. Gene expression of proliferation markers Mki67, Mybl2, Ccnb1 and Pcna in hFOB 1.19 cells in response to supplementation of VLC-PUFA or DHA (1 and 2 uM). Total RNA was extracted and qPCR analysis for gene expression performed. A: Harvested day 2 after seeding (approximately 24 hours with supplementation of substrates). B: Harvested on day 3 after seeding (approximately 48 hours with supplementation). Comparisons were conducted using one-way ANOVA and Student’s t test. Data are presented as the mean ± SEM of three trials (n=3), where each trial consisted of three replicates per test dose, except for one of the trials, in which the control group consisted of six replicates. The significance level was set to P < 0.05.

Table S1. Fatty acid composition of salmon feed.

|  | Diet groups | | | | |
| --- | --- | --- | --- | --- | --- |
|  | Ctr | L-VLC | I-VLC | MH-VLC | H-VLC |
| 14:0 | 6.01 | 5.85 | 5.74 | 5.42 | 5.44 |
| 15:0 | 0.11 | 0.11 | nd | 0.38 | 0.11 |
| 16:0 | 12.56 | 12.46 | 12.43 | 12.02 | 12.06 |
| 17:0 | 0.18 | 0.17 | 0.73 | 0.17 | 0.15 |
| 18:0 | 1.43 | 1.45 | 1.32 | 1.35 | 1.36 |
| 20:0 | 0.16 | 0.00 | 0.14 | 0.15 | 0.15 |
| 24:0 | 0.17 | 0.18 | 0.16 | 0.21 | 0.19 |
| **Sum of SFAs** | **20.64** | **20.22** | **20.53** | **19.70** | **19.48** |
| 14:1 *n*-5 | 0.32 | 0.35 | 0.74 | 0.34 | 0.28 |
| 15:1 | 0.19 | 0.18 | 0.19 | 0.21 | 0.16 |
| 16:1 *n*-7 | 4.63 | 4.16 | 4.07 | 3.98 | 4.31 |
| 16:1 *n*-5 | 0.42 | 0.41 | 0.46 | 0.41 | 0.12 |
| 17:1 *n*-7 | 0.34 | 0.33 | 0.43 | 0.33 | 0.29 |
| 18:1 *n*-7 | 1.74 | 1.79 | 1.69 | 1.72 | 1.73 |
| 18:1 *n*-9 | 11.90 | 11.65 | 10.96 | 11.23 | 11.30 |
| 18:1 *n*-11 | 0.45 | 0.08 | 0.41 | 0.41 | 0.22 |
| 19:1 | 0.14 | 0.12 | 0.13 | 0.14 | 0.14 |
| 20:1 *n*-7 | 10.68 | 10.63 | 9.23 | 9.63 | 10.59 |
| 20:1 *n*-11 | 2.32 | 2.30 | 2.21 | 2.24 | 2.25 |
| 22:1 *n*-7 | 0.49 | 0.49 | 0.46 | 0.48 | 0.48 |
| 22:1 *n*-9 | 1.78 | 1.87 | 0.84 | 1.51 | 1.56 |
| 22:1 *n*-11 | 15.99 | 15.85 | 14.34 | 14.80 | 14.90 |
| 24:1 *n*-9 | 0.97 | 1.05 | 1.08 | 1.20 | 1.22 |
| **Sum of MUFAs** | **52.37** | **51.25** | **47.25** | **48.63** | **49.55** |
| 16:2 *n*-6 | 0.35 | 0.33 | 0.30 | 0.33 | 0.32 |
| 18:2 *n*-6 | 4.02 | 4.07 | 4.26 | 4.47 | 4.50 |
| 18:3 *n*-6 | 0.13 | 0.13 | 0.13 | 0.12 | 0.12 |
| 20:2 *n*-6 | 0.22 | 0.21 | 0.19 | 0.19 | 0.19 |
| 20:4 *n*-6 | 0.31 | 0.33 | 0.34 | 0.36 | 0.37 |
| 22:4 *n*-6 | 0.32 | 0.32 | 0.30 | 0.29 | 0.30 |
| **Sum of *n*-6 PUFAs** | **5.34** | **5.39** | **5.52** | **5.76** | **5.81** |
| 16:2 *n*-3 | 0.32 | 0.31 | 0.29 | 0.29 | 0.25 |
| 20:4 *n*-3 | 0.64 | 0.47 | 1.21 | 0.86 | nd |
| 20:5 *n*-3 | 5.50 | 5.60 | 6.78 | 6.49 | 6.46 |
| 22:5 *n*-3 | 0.95 | 1.08 | 1.13 | 1.31 | 1.32 |
| 22:6 *n*-3 | 9.20 | 9.43 | 8.80 | 9.43 | 9.48 |
| **Sum of *n*-3 PUFAs** | **17.81** | **18.07** | **19.36** | **19.56** | **18.71** |
| **Sum EPA/DHA** | **14.71** | **15.03** | **15.58** | **15.92** | **15.95** |
| **Sum FA** | **98.93** | **98.79** | **98.71** | **98.73** | **98.23** |

_Data are presented as percentage of total fatty acids._

_Ctr, control; L-VLC, Low-VLC; I-VLC, Intermediate-VLC; MH-VLC, Medium-High-VLC; H-VLC, High-VLC; ND, not detected._
